# Recent innovation in benchmark rates (BMR): evidence from influential factors on Turkish Lira Overnight Reference Interest Rate with machine learning algorithms

**DOI:** 10.1186/s40854-021-00245-1

**Published:** 2021-06-12

**Authors:** Özer Depren, Mustafa Tevfik Kartal, Serpil Kılıç Depren

**Affiliations:** 1 Yapı Kredi Bank, Istanbul, Turkey; 2 Borsa İstanbul Financial Reporting and Subsidiaries Directorate, Reşitpaşa Mahallesi Borsa İstanbul Caddesi, No: 4, 34467 Istanbul, Turkey; 3 Department of Statistics, Yildiz Technical University, Istanbul, Turkey

**Keywords:** Benchmark rate, Determinants, Machine learning algorithms, Turkey, C40, E43, E44, G12

## Abstract

Some countries have announced national benchmark rates, while others have been working on the recent trend in which the London Interbank Offered Rate will be retired at the end of 2021. Considering that Turkey announced the Turkish Lira Overnight Reference Interest Rate (TLREF), this study examines the determinants of TLREF. In this context, three global determinants, five country-level macroeconomic determinants, and the COVID-19 pandemic are considered by using daily data between December 28, 2018, and December 31, 2020, by performing machine learning algorithms and Ordinary Least Square. The empirical results show that (1) the most significant determinant is the amount of securities bought by Central Banks; (2) country-level macroeconomic factors have a higher impact whereas global factors are less important, and the pandemic does not have a significant effect; (3) Random Forest is the most accurate prediction model. Taking action by considering the study’s findings can help support economic growth by achieving low-level benchmark rates.

## Introduction

Interest rates can be described as the price of money. They function as determining instruments of the cost or value of money in economies (Tumwine et al. 2018; Lyashenko and Mercurio 2019). Depending on the functions, interest rates are among the most important economic and financial indicators. Therefore, interest rates have the potential to affect the indicators while the indicators may affect interest rates.

Changes in interest rates negatively affect financial markets and all economic actors. For example, increasing interest rates affect investments negatively (Lin et al. 2018). Similarly, increasing interest rates causes a decreasing effect on economic activities and growth. Interest rates are important in terms of price, macroeconomic, and financial stabilities. Therefore, countries desire to have low interest rates, considering their significant role. According to the role and importance of interest rates in economies, the European Union (EU) area has been applying negative interest rates. Similarly, Turkey has implemented a negative interest rate until recently. However, the desired stimulus effect cannot be achieved with the negative interest rate practice in the EU and Turkey.

Monetary, fiscal, and macro-prudential policies affect interest rates either positively (decreasing) or negatively (increasing) depending on the decisions taken. Changes and volatility of interest rates can change stock markets, foreign investments, credit expansion, credit growth, and economic growth by affecting uncertainty (Gözgör et al. 2019). Additionally, savings, consumption, and unemployment are related to interest rates. Therefore, the level and behavior of interest rates are crucial for the stability and development of countries.

‘Interest rate’ is used as a generic term. However, there are various types of interest rates in economies. Central Bank policy rates, credit interest rates, deposit interest rates, interbank interest rates, Treasury bond interest rates, Eurobond interest rates, and London Interbank Offered Rate (LIBOR) are interest rates (Dinçer et al. 2019; Salim 2019; Kartal 2020a). Each of these has a different function in financial markets and economies and can have different rate levels in the same term. While most types of interest rate are related to national activities, few are related to the international arena like LIBOR.

LIBOR is a type of interest rate used mostly in international lending and borrowing activities, capital flows, and fund flows. Additionally, LIBOR is used for lending without collaterals (Johannes and Sundaresan 2007). Besides, LIBOR is calculated based on the bid and ask prices quoted by investment banks included in the LIBOR system. LIBOR is not calculated based on the transactions realized in an organized market. There have been discussions about LIBOR’s reliability because of this structure, because LIBOR can be manipulated by banks providing misleading quotations. Some regulations have even fined the banks involved in the formation of LIBOR. Additionally, LIBOR is criticized for not reflecting counterparty risk very clearly (Tokic 2018).

LIBOR will be retired at the end of 2021 due to the contributions of negative arguments and developments and scandals in 2012 (Kalgreen 2019; European Union (EU) 2021a). Some countries have been working on their national benchmark rates (BMR) to meet their needs to replace LIBOR. Accordingly, various legislations have been made by regulatory bodies (EU 2016), and some additional legislation on benchmark rates has been in the negotiation process (EU 2021b).

While some countries, including Turkey, have announced national benchmark rates (Bank for International Settlements (BIS) 2021; Borsa İstanbul (BIST) 2021a; International Swaps and Derivatives Association (ISDA) 2021a), some others have been working on the development of their BMR, which is required and essential for the period after LIBOR is retired. In the Turkey example, BIST announced Turkish Lira Overnight Reference Interest Rate (TLREF) as a new financial instrument in 2019. TLREF is calculated based on the transactions realized each day in BIST repo-reverse repo market.

Considering all the information above, having a national benchmark rate and sustaining a low-level of TLREF is significant due to its influence on the financing capacities of banks to in turn affect economic development and growth. In this context, as a new financial innovation instrument, examining the effects of global and country-level macroeconomic factors on TLREF can be empirically beneficial. Hence, some policy recommendations can be proposed to regulatory bodies based on analysis of these factors so that Turkey can decrease the negative effects of the influential determinants on TLREF and can be ready for replacing LIBOR with TLREF in financing activities. Hence, it stimulates economic activities and growth via increased borrowing, lending, and TLREF-based securities issuance capacity of Turkish banks.

This study examines the determinants of TLREF as a new financial instrument announced in the context of transition to benchmark rates after LIBOR retirement. This is done by employing machine learning algorithms to reveal the important variables (including global and country-level macroeconomic determinants and the pandemic) while focusing on an emerging market example (i.e., Turkey) to understand how to determine the measures through which countries can achieve low-level benchmark rates. The dataset collected for this objective is drawn from daily data between December 28 2018, and December 31, 2020. The results of the study show that the most influential factors on TLREF are the amount of securities bought by the Central Bank followed by emission amount, gold prices, United States Dollar (USD)/TL foreign exchange rates (FX), BIST main index (XU100), the volatility index, credit default swap (CDS) spreads, Morgan Stanley Capital International (MSCI) emerging market index, respectively. Moreover, the COVID-19 pandemic does not affect TLREF throughout the periods examined. The results of the analysis show the crucial effects of country-level macroeconomic factors on TLREF.

Although there is a transition period, and guidelines and rules have been published by regulatory authorities for the post-LIBOR period (preparatory works), the nexus between benchmark rates, economic factors (i.e., global and country-level), and the pandemic is unclear. This issue has not been explored comprehensively yet. We obtain some insight into preparing for such an issue in the attempt to fill one of the gaps in the existing literature. The study contributes to the literature in certain ways. Although there is a study examining TLREF conceptually (Kartal 2019a), to the best of our knowledge, there is no study that examines TLREF empirically in the literature. Therefore, the study’s main contribution is to examine and reveal the determinants of TLREF by using global and country-level macroeconomic indicators, while also considering the pandemic. This study focuses on Turkey’s benchmark rate through machine learning algorithms, which have high prediction capacity and regression. In this context, the Random Forest algorithm has the most accurate prediction model, followed by Support Vector Machines, Ordinary Least Square (OLS), and k-Nearest Neighbors algorithms. This study recommends some policy implications for low-level TLREF by considering the analysis of the results.

The remainder of this study is organized as follows. “Conceptual framework” section  briefly presents the conceptual framework, while a literature review is provided in “Literature review” section. “Data, variables, and methodology” section describes the data, determinants used in the analysis selected from the literature, and the methodology. “Empirical results” section presents the empirical results, findings-based discussion, and policy implications. Finally, the conclusions are discussed in “Conclusion” section.

## Conceptual framework

A benchmark can be defined as “an index (statistical measure), calculated from a representative set of underlying data, that is used as a reference price for a financial instrument or financial contract, or to measure the performance of an investment fund” (EU 2016). A benchmark rate shows the average interest rate in a specific economy (Kartal 2019b). With this definition and specification, BMR is used in various products such as forward, swap option, float rate bonds or binds, mortgage loans, and hybrid financial instruments (Chan 2011). BMR importantly reflects the realities in financial markets.

Previously, various term rates (e.g., 1 month, 3 months) were determined by fixing in advance, and overnight rates were compounded for different tenors by fixing in arrears (Lyashenko and Mercurio 2019; ISDA 2021b). Recently, the calculation method for benchmark rates has changed. Benchmark rates, which replace the LIBOR after the end of 2021 as fallback rates, have been constructed based on risk-free overnight rates mainly by Central Banks or countries’ stock exchanges (Bank of England (BoE) 2021; ISDA 2021b). Additionally, benchmark rates are constructed based on real transactions rather than fixing or quotations, important for new generation benchmark rates. Moreover, the spread between fallback rates and adjusted rates is very low. For example, overnight USD LIBOR is 0.08313, whereas the adjusted overnight reference rate (SOFR) is 0.08, showing a 0.00313 spread. Similarly, overnight GBP LIBOR is 0.05290, whereas the adjusted overnight reference rate (SONIA) is 0.05, showing a 0.00290 spread (Bloomberg 2021). These figures prove that new benchmark rates can be replaced with LIBOR.

With the transition process to the period after LIBOR, countries (regions) have been making efforts to announce national benchmark rates. In this context, some countries (regions) like the USA, UK, Euro Area, Switzerland, Japan, Australia, Canada, and Hong Kong announced benchmark rates as Secured Overnight Financing Rate (SOFR), Sterling Overnight Index Average (SONIA), Euro Short Term Rate (ESTR), Swiss Average Rate Overnight (SARON), Tokyo Overnight Average Rate (TONA), Australian Interbank Overnight Cash Rate (AONIA), Canadian Overnight Repo Rate Average (CORRA), and Hong Kong Dollar Overnight Index Average (HONIA), respectively (BIS 2021; ISDA 2021a). Moreover, Turkey has advertised TLREF as BMR, a new financial instrument recently developed by BIST. Turkey tried another benchmark rate named Turkish Lira Reference Interest Rate (TRLIBOR) in the past. However, it was not successful because banks did not use this rate, and there were not many transactions based on the TRLIBOR rate. The 1-week repo interest (policy) rate of the Central Bank is not enough sometimes. Thus, the policy rate may not reflect the funding rate in the market. Therefore, TLREF was developed and announced as a new benchmark rate to meet Turkey’s BMR needs in 2019. Hence, TLREF is used in borrowing and lending activities, derivative products, bonds and bills, and other financial instruments (BIST 2021a).

**Fig. 1:**
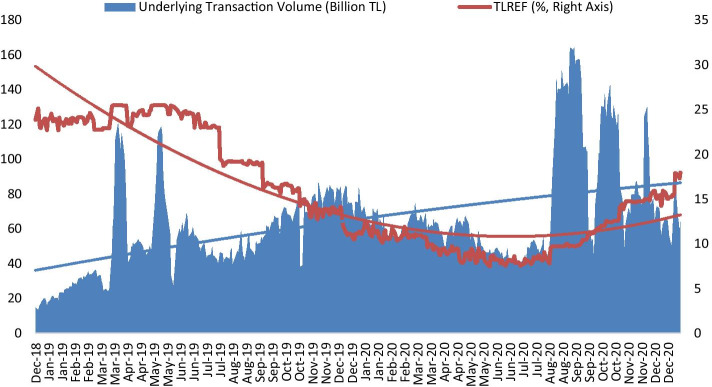
The development of TLREF and underlying transaction volume. *Source*: BIST (2021a)

TLREF is calculated based on the transactions realized in BIST repo-reverse repo market, which is significant for the BMR requirement. In the calculation, certain types of transactions (canceled transactions, special notifications, transactions settled outside of Takasbank, transactions from itself) are excluded. Additionally, the bottom and top 15% of transactions are excluded (BIST 2021a). TLREF was announced first on June 17, 2019, and it included data from December 28, 2018, the date of the consolidation of repo markets and hence consolidation of liquidity in BIST. Figure 1 shows the development of TLREF.

**Fig. 2:**
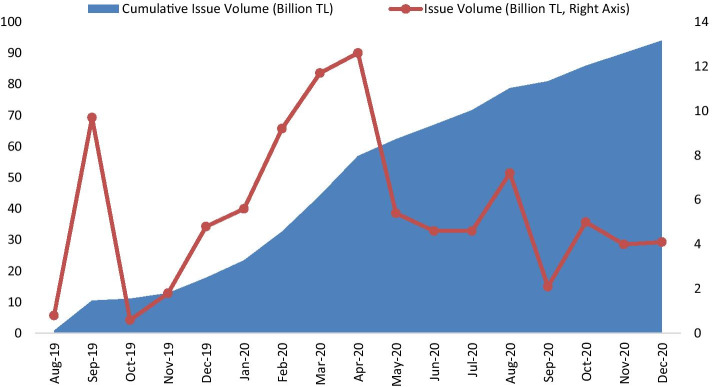
The development of TLREF-based securities (billion TL). *Source*: Authors’ collections (Public Disclosure Platform PDP 2021)

According to Fig. 1, TLREF has a decreasing trend on average. Additionally, there is a significantly increasing underlying transaction volume. Hence, it can be said that TLREF does not have shortcomings, such as the quotation-based determination that LIBOR has. Therefore, TLREF is a highly reliable BMR for Turkey and other parties. However, with the announcement of TLREF, financial institutions have to issue TLREF-based securities. Figure 2 shows the development of TLREF-based securities (bond and bills).

Figure 2 presents that securities issues began in August 2019. After this time, the cumulative issue volume of TLREF based securities has constantly increased. The new issuance of TLREF-based securities has a volatile trend depending on the funding needs of related parties, mainly banks, other financial institutions, the Turkish treasury, and some corporate firms. In summary, the amount of TLREF based issues has reached TL 94 billion as of 2020 year-end. Additionally, this volume may continue to increase with the replacement of LIBOR.

With the introduction of TLREF, Turkey has been preparing to replace LIBOR with TLREF. However, the level of TLREF would be very important in the forthcoming period as it will directly affect the financial sources of Turkish banks via borrowing activities and securities.

## Literature review

There are limited but growing studies in the literature examining the nexus between BMR and determinants. For example, Chan (2011) examines BMR in Hong Kong between January 2007 and June 2010 by using a cointegration test and concludes that corporations can lower the interest rate risks using LIBOR rather than Hong Kong Interbank Offered Rate (HIBOR) and Singapore Interbank Offered Rate (SIBOR). Kanlı (2012) studied the BMR in Turkey between January 2007 and July 2012 and determined that TRLIBOR is the best indicator reflecting market expectations well in the short run. Additionally, Terzioğlu (2013) reviewed the BMR in Turkey between January 2006 and May 2012 by applying the cointegration test and concluded that foreign trade deficit and the increase in internal debt volume cause the increase in BMR. Akkaya (2018) studied the factors influencing TRLIBOR in Turkey between January 2003 and June 2017 using Auto-Regressive Distributed Lag (ARDL) bounding test and defined that real effective FX and change in consumer prices index significantly affect BMR. Moreover, Kim and Shi (2018) worked on BMR in China between 1987 and 2013 using quarterly data and probit models. They determined that inflation and money growth were the main factors. Regarding TLREF, the only examination performed is by Kartal (2019b) on a conceptual base.

Additionally, the nexus between different types of interest rates and determinants has great importance in the literature. There are various determinants like budget deficit (Mukhtar and Zakaria 2008), current account balance (Jammazi et al. 2017; Dinçer et al. 2019), economic growth (Holston et al. 2017; Obeng and Sakyi 2017; Shaukat et al. 2019), financial sector development (Egert et al. 2007), foreign trade (Gupta and Goyal 2015), inflation (Muinhos and Nakane 2006; Ratti and Vespignani 2016), public debt (Tumwine et al. 2018), reserves (Gupta and Goyal 2015), and unemployment (Hol 2006; Taylor and Wieland 2016; Mitchell and Pearce 2017; Shapiro 2018), etc. However, these indicators are not included in this study, considering that these variables are announced on a monthly, quarterly, or yearly basis, whereas TLREF is announced daily. Considering the availability of data for independent determinants, three global and five country-level macroeconomic determinants and the pandemic are selected to analyze TLREF.

In the global determinant group, gold prices, MSCI emerging market index, and the volatility index (VIX) are included. Gold prices can affect TLREF in a similar (positive) or opposite (negative) direction depending on countries’ nature related to gold activities. Additionally, as an important volatility indicator, VIX can positively affect emerging countries up to a point. However, the MSCI emerging market index, which affects investment flows to emerging countries like Turkey, may negatively affect TLREF.

CDS spreads, FX, stock market index, amount of securities bought, and amount of money issued by Central Bank are considered in the country-level macroeconomics determinants group. CDS spreads have a relationship with interest rates. For example, Kartal (2020b) defines a positive relationship between the interest rate (Central Bank weighted average cost of the fund) and CDS spreads for Turkey using multivariate adaptive regression splines. Additionally, the relationship between CDS spreads, and different type of interest rates (e.g., interest rates of global fixed income convertible bonds, reinvestment rate, spot rate, Treasury bond interest rates) are researched in some studies like Alexander and Kaeck (2008), Galil and Soffer (2011), Galil et al. (2014), Hibbert and Pavlova (2017) and Yang et al. (2018) and a positive relationship is defined. Considering these studies, a positive effect of CDS spreads expected on TLREF. Five-years CDS USD spreads are selected as determinants as this maturity has the most liquidity (Hasan et al. 2016).

FX is related to interest rates. Various studies (Hol 2006; Paramati and Gupta 2013; Gupta and Goyal 2015; Maitra 2017; Obeng and Sakyi 2017; Kartal et al. 2018; Gopinathan and Durai 2019; Kartal 2019b) have researched the effects of FX on the different type of interest rates (e.g., credit interest rate) in various countries (Italy, selected countries (Norway, Sweden, Denmark), Sri Lanka, India, Ghana, Turkey). A positive relationship between FX and interest rates is generally found in these studies. However, FX can negatively affect interest rates while the investment preferences of investors are directed to FX rather than interest rates. By considering these studies, either positive or a negative effect of FX can be seen on TLREF. USD/TL FX is selected as a determinant because it is popular in Turkey.

Additionally, indicators related to stock markets are used as a determinant for examining interest rates. Wong et al. (2006) worked on Singapore and the USA for the period from January 1982 to December 2012 by using cointegration and causality tests and determined that stock prices of Singapore are related to the interest rate in the long run; however, a similar relationship is not valid for the USA. Jammazi et al. (2017) used the USA sample and determined that stock returns are one of the driving forces of interest rates in the USA. Tursoy (2019) studied Turkey and defined that stock prices are negatively correlated with interest rates. Hence, a negative effect of the stock market index is expected on TLREF. XU100 index is selected as a determinant as this is the main index in Turkey.

Additionally, the money supply is a determinant that should be considered in interest rate analysis. In this context, a different type of money supply indicator (emission volume, M2 volume, total money supply) is examined by researchers (Onanuga and Shittu 2010; Gupta and Goyal 2015; Kartal 2019b) in countries (Europe, Nigeria, India, Turkey). A negative relationship between money supply and interest rates is found generally in these studies. A negative effect of the money supply is expected on TLREF. The amount of money issued by the Central Bank is selected as a determinant because this is the main money supply indicator.

In addition to these four country-level factors, the amount of securities bought by the Central Bank can affect the interest rate. Therefore, TLREF is calculated in the repo-reverse repo market where Treasury bonds are traded. The amount of securities bought by the Central Bank increases Treasury bonds bought and sold and restricts the outstanding volume of Treasury bonds in the market. Therefore, this is considered in the analysis as a determinant. Also, the effect of the determinants is expected to be negative.

Moreover, a black swan case called the COVID-19 pandemic occurred at the end of 2019 (World Health Organization (WHO) 2021). This unprecedented case has affected countries worldwide, economic and financial markets, and indicators (Goodell 2020; Rizwan et al. 2020). The pandemic’s presence in the analysis was defined on March 11, 2020, for the first time in Turkey (Ministry of Health of Turkey (MHT) 2020). Hence, a total of nine determinants are selected for analysis in this study.

As BMR are relatively new, they can be observed in new financial studies with new functions, which replace LIBOR. By focusing on global and country-level macroeconomic determinants that may influence the interest rate, with the data including the entire period since the announcement of BMR, applying machine learning algorithms can contribute to the literature.

## Data, variables, and methodology

### Data sources

The daily data between December 28, 2018, and December 31, 2020, was used in the study. Data for TLREF and independent determinants were gathered from BIST (2021b) and Bloomberg (2021), the Central Bank of the Republic of Turkey (CBRT 2021), and MHT (2020) to analyze the determinants of the TLREF.

### Variables

The dependent variable is TLREF. According to the literature review, there is a nexus between interest rates and various determinants. Independent variables used in the analysis are defined from the literature review by considering data availability and grouped as global or country-level factors. Table 1 summarizes the variables.

**Table 1:** Independent variables

Group	Variable	Symbol	Description	Effect
Global	Gold prices	GOLD	Gold prices per ounce (USD)	+, −
	MSCI emerging market index	MSCI_EM	MSCI emerging market index	−
	VIX index	VIX	Chicago board options exchange volatility index	+, −
Country-level macroeconomic	CDS spreads	CDS	5-years CDS spreads (USD)	+
	BIST main index	XU100	Trading day closing value	−
	USD/TL FX	USDTL	USD/TL FX	+, −
	CBRT securities	CBRT_SEC	Amount of Securities Bought By CBRT	−
	Emission amount	EMISSION	Amount of money issued by CBRT	−
Other	COVID-19	COVID	1: If the pandemic exists; 0: otherwise	+, −

A positive (+) relationship means that TLREF increases when independent variables increase

A negative (−) relationship means that TLREF decreases when independent variables increase

### Methodology

#### Data preprocessing

Daily data are collected from different sources. All data are combined after considering missing values in the period.

**Fig. 3:**
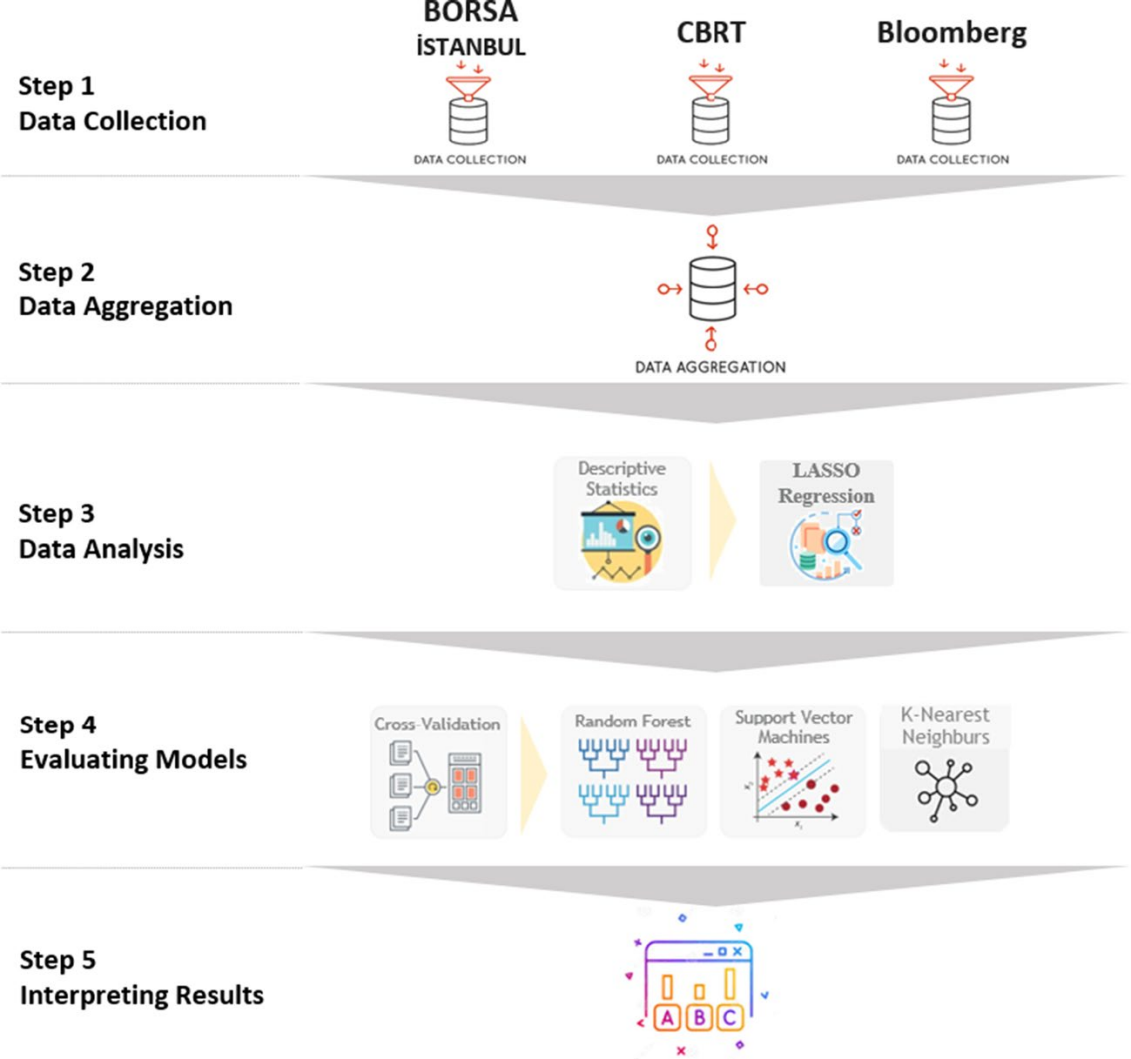
The process of the methodology in the research. *Source*: Authors’ construction

The model is constructed in five steps, data collection, data aggregation, data analysis, evaluating models, and interpreting results. The flowchart of the methodology is detailed in Fig. 3.

First, the data collection step of the methodology consists of obtaining data from different data sources that affect TLREF. The second step is to aggregate the datasets to generate one combined dataset. In the third step, descriptive statistics and Least Absolute Shrinkage and Selection Operator (LASSO) Regression output are given. In the fourth step, three different machine learning algorithms, which are Random Forest, Support Vector Machines, and k-Nearest Neighbors, are performed with a cross-validation approach. In the final step, results obtained from the models are evaluated.

#### Model building

In the last decade, researchers have frequently used supervised or unsupervised machine learning algorithms to overcome real-life problems in different areas such as banking, finance, economics, education, and medicine. In these areas, the frequently used algorithms are Random Forest, Support Vector Machines, k-Nearest Neighbors, Naïve Bayes, and Neural Networks (Kou et al. 2014). In the literature, many studies use machine learning algorithms (and deep learning algorithms) with a cross-validation approach to predict the factors affecting the dependent variable in the time series data (Kou et al. 2019, 2021). Machine learning algorithms with the classical repeated cross-validation approach are used to determine the influencing factors affecting TLREF (Parmezan et al. 2019; Shen et al. 2020; Kumar et al. 2021; Zhang et al. 2021).

The Random Forest algorithm, proposed by Breiman in 2001, is based on the bagging learning method. The process of the algorithms is as follows (Schonlau and Zou 2020):

1. Subsamples with size n are selected randomly (n_tree_), indicating the number of trees constructed.
2. m_try_ parameter determines the number of predictors included for each split of the node.
3. For each split, the best predictors are selected to prune the tree.
4. The target variable is estimated by aggregating the results of each tree.

The Support Vector Machines approach proposed in 1992 for classification and regression problems is older than the Random Forest. The mathematical background of this approach is based on minimizing the regression error using the cost function (e-intensive loss function). The minimization problem is described as follows (Law and Shawe-Taylor 2017):

1 $$\begin{gathered} objective: \mathop {\min }\limits_{w,b} \frac{1}{2}w^{2} + C\mathop \sum \limits_{i = 1}^{n} \left( {\xi_{i}^{ + } + \xi_{i}^{ - } } \right) \hfill \\ subject: \hfill \\ y_{i} - w.x_{i} - b \le \varepsilon + \xi_{i}^{ + } \hfill \\ w.x_{i} + b - y_{i} \le \varepsilon + \xi_{i}^{ - } \hfill \\ \xi_{i}^{ + } , \xi_{i}^{ - } \ge 0 \forall i \hfill \\ \end{gathered}$$

The first term of the objective function represents the regularization constraint to prevent over-fitting and the second term is the ε-insensitive loss function. $\xi_{i}^{ + } , \xi_{i}^{ - }$ represent the slack variables in the optimization problem. In Eq. 1, y, x, and b represent the dependent variable, independent variables, and the error term, respectively. The term w is the weight parameter of the model.

The k-Nearest Neighbors algorithm, based on the calculation of distance measures, is proposed by Bentley in the early 1970s. Although different distance measures can be used in this analysis, Euclidean distance, Manhattan Distance, and Minkowski distance are frequently used for regression or classification problems. The algorithm steps are as follows (Khun and Johnson 2013):

1. Determine the k value, which is the nearest observation to be used in the distance calculation.
2. Select suitable distance measure based on the dataset.
3. Predict the target variable according to the selected distance measure.

#### Model testing and cross-validation

Separation of the dataset into training and testing datasets is the most crucial step of machine learning modeling. The training dataset is used to enhance the model, while the test dataset is used to test the model results to prevent overfitting or under-fitting problems. A k-fold with t-repeat cross-validation is used in the machine learning algorithms to improve the model accuracy. In this study, k and t are set as 10 and 5, respectively, for obtaining a robust model. The steps for this process are as follows (Kucheryavskiy et al. 2020):

1. Dataset has separated into k equal-sized subsamples.
2. k-1 sub-samples are used as training datasets, and one sample is used as a testing dataset.
3. k estimations are used for reaching a single estimation.
4. Steps 1 to 3 are repeated t times to produce more robust results.

## Empirical results

### Descriptive statistics

Before creating a machine learning model to estimate TLREF, the LASSO regression approach, a feature selection analysis, is performed to determine the most important factors affecting TLREF. As a result of the LASSO regression process, oil prices are excluded from the analysis because the correlation between oil prices and TLREF is not statistically significant.

**Fig. 4:**
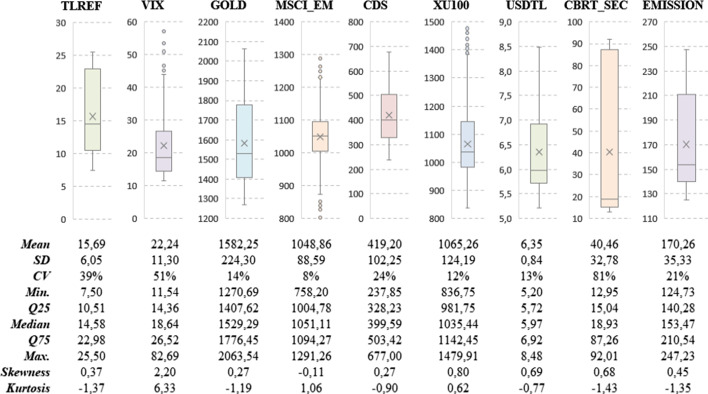
The box and whisker plot and descriptive statistics

Box and Whisker Plot (which provides the basic characteristics of a variable) of each variable is given in Fig. 4.

In Fig. 4, the average, median, and standard deviation (SD) of TLREF is 15.69, 14.58, and 6.05, respectively. As VIX, MSCI_EM, and XU100 have several outliers, the Coefficient of Variation (CV) of MSCI_EM is lower than 10%, while the CV value of VIX and CBRT_SEC is higher than 50%. Furthermore, skewness and kurtosis statistics of VIX are significantly higher than others.

Furthermore, Brock–Dechert–Scheinkman (BDS) test is performed to check the non-linearity structure of variables. The results of the BDS test are presented in “Appendix”. According to the BDS test results, the null hypothesis is rejected. Hence, the results indicate the non-linearity of the variables.

### The performance of the algorithms

R^2^, Root Mean Square Error (RMSE), and Mean Absolute Error (MAE), the goodness of fit criteria, are used to determine the best algorithms that can predict level values of the TLREF. The goodness of fit criteria is given in Table 2 for Random Forest, Support Vector Machines, and k-Nearest Neighbors algorithms. Additionally, the OLS method is performed to compare the results between machine learning algorithms and the traditional approach.

**Table 2:** The goodness of fit criteria of the models

Algorithm	R square (%)	RMSE	MAE
Random forest	99.1	0.610	0.415
Support vector machines	94.5	1.505	1.164
OLS regression	91.2	1.804	1.375
k-nearest neighbors	63.6	3.822	2.684

Based on Table 2, R^2^ values of Random Forest and Support Vector Machines algorithms are high. However, the goodness of fit criteria of the k-Nearest Neighbors algorithms is not satisfactory for predicting TLREF. The Random Forest algorithm has the highest R^2^ and lowest RMSE and MAE values than the other algorithm. Therefore, the Random Forest algorithm details are used for interpretation. Although the R^2^ value of the OLS method is 91.2%, the impact of some factors is not statistically significant. Thus, this can be strong evidence that parametric model assumptions are not met in OLS. Since multicollinearity and other assumptions should be tested to continue the OLS analysis, it is preferred to use machine learning approaches with no strict assumptions on the data and distributions.

### Analysis results

**Fig. 5:**
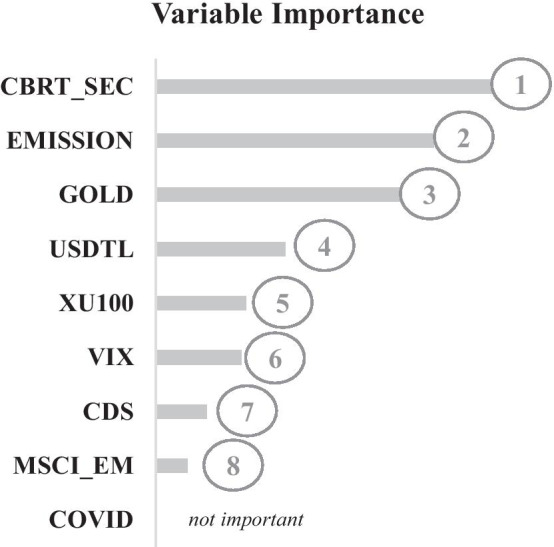
Variable importance analysis of random forest algorithm. 1 shows the most important variable, whereas 8 implies the least important variable

Variable importance analysis is performed to measure the effects of the independent determinants. Variable importance for the Random Forest algorithm is given in Fig. 5.

According to the variable importance analysis, it is determined that amount of securities bought by CBRT (CBRT_SEC) is the most important factor affecting TLREF, followed by the amount of money issued by CBRT (EMISSION), gold prices (GOLD), FX (USD/TL FX), main stock market index (XU100), the volatility index (VIX), CDS spreads (CDS), and MSCI emerging market index (MSCI_EM). COVID is determined as the factor that has no significant impact on TLREF.

**Fig. 6:**
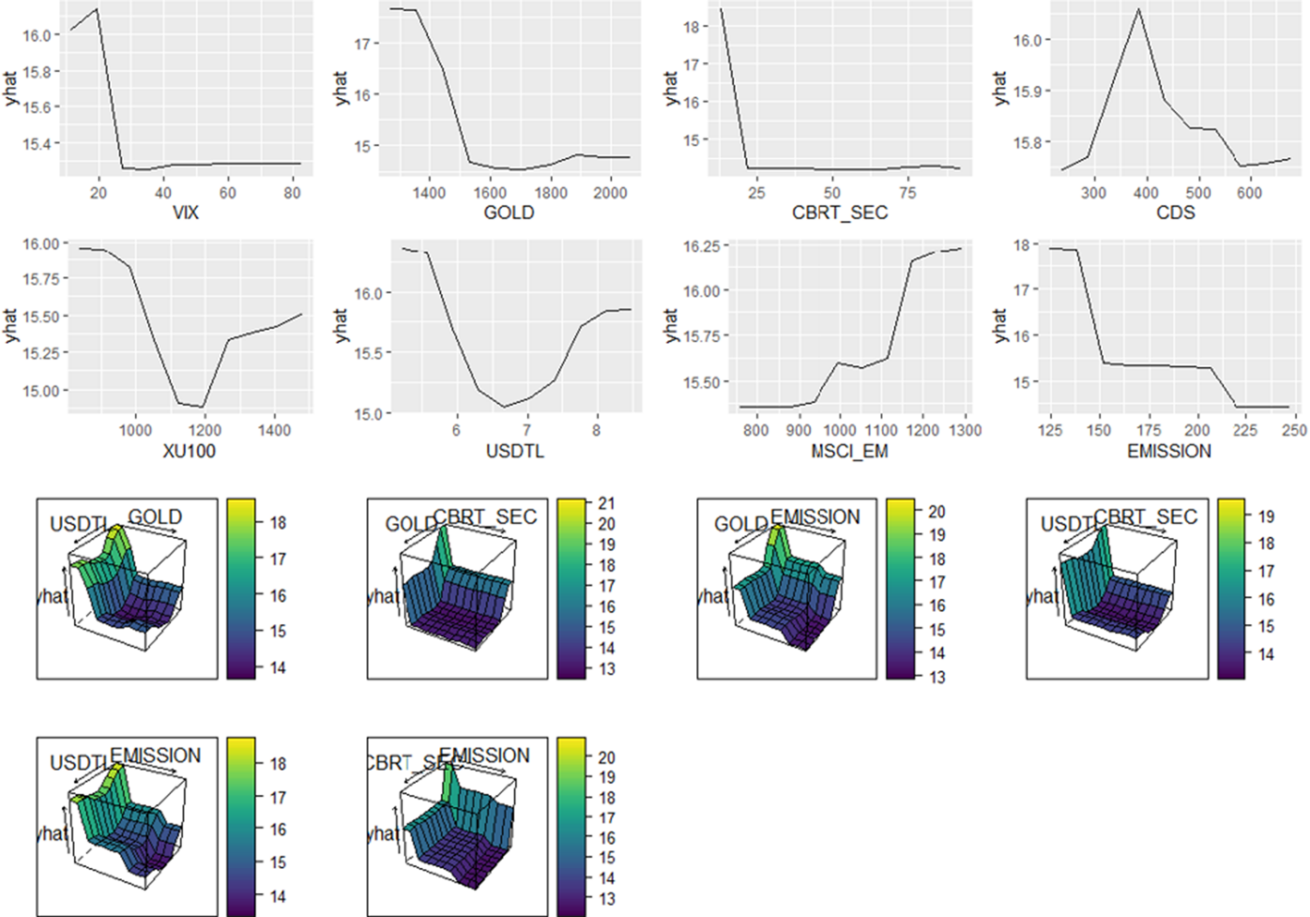
Thresholds of the independent determinants by TLREF

In addition to variable importance analysis, thresholds crucial to measuring the impact of the independent determinants on TLREF are also analyzed. In Fig. 6, thresholds of the independent determinants by predicted values of TLREF are given.

Figure 6 shows that VIX should be higher than 30 to reduce TLREF. Similarly, the critical thresholds for GOLD, CBRT_SEC, and EMISSION are 1.500, 20, and 150, respectively. This means that GOLD must be over 1.500, CBRT_SEC over 20, and EMISSION over 150 to keep TLREF low. However, CDS should be under 350 to reduce the TLREF. XU100 and USDTL should be between 1.100–1.200 and 6.5–7.5, respectively, to keep TLREF at low levels. Also, MSCI_EM should be lower than 950 to reduce the LREF.

Interaction effects between independent determinants are analyzed in the analysis. Based on the interaction effect of GOLD and USDTL, the optimum scenario is that GOLD should be higher than 1.500 while USDTL should be between 6.5 and 7.5 to reduce TLREF. Additionally, interaction effects of GOLD and CBRT_SEC, GOLD and EMISSION, USDTL and CBRT_SEC, USDTL and EMISSION, and CBRT_SEC and EMISSION have a statistically significant impact on TLREF.

**Fig. 7:**
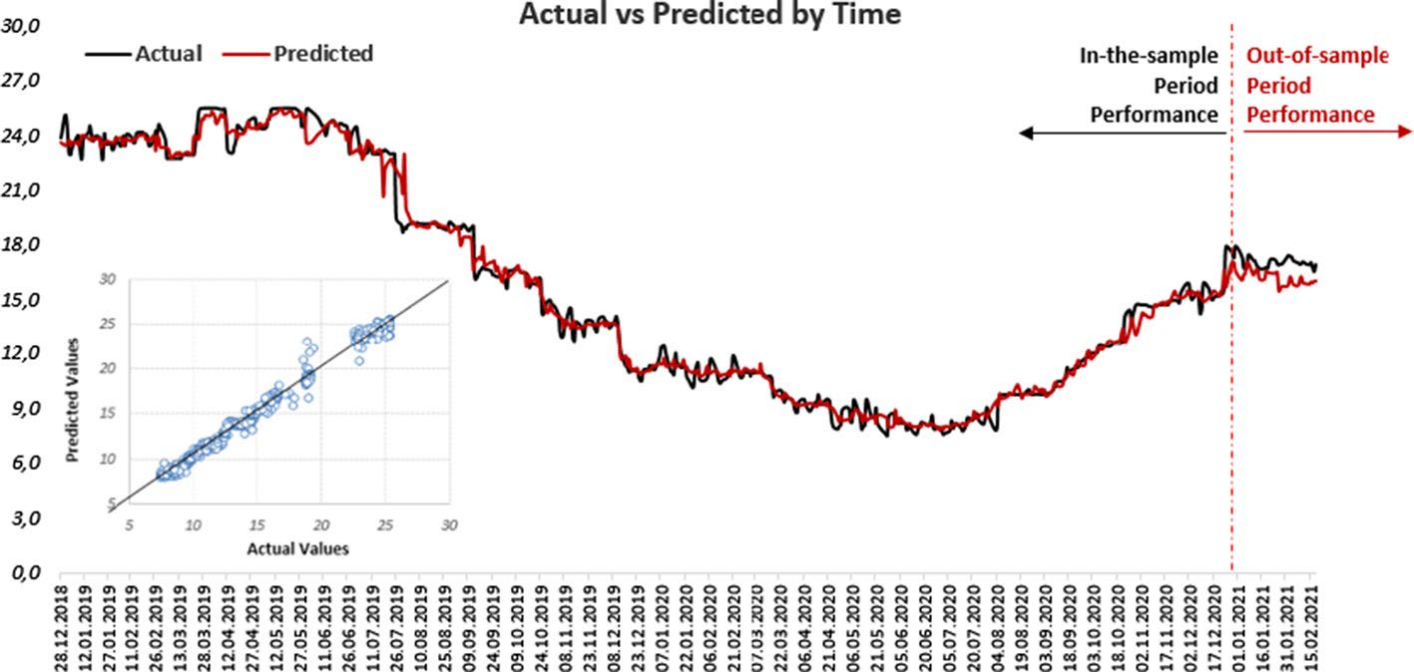
Actual and predicted values of TLREF

The actual and predicted values of the TLREF, predicted with the Random Forest algorithm, are given in Fig. 7.

Model performance of both in-the-sample period and out-of-sample period is measured in this study. The actual and predicted values of TLREF are quite close to each other in both periods. Although the prediction model can predict sudden increases or decreases very well in the in-the-sample period, the predicted values of TLREF in the out-of-sample period are slightly lower than the actual values after January 31, 2021. In comparing actual and predicted values, it is assumed that in a graph where the actual values on the horizontal axis and the predicted values on the vertical axis, all data points are expected to be on (or close to) a 45° line. In this way, it is possible to visualize how close the predicted values are to actual values. In our study, all data points are almost placed on the 45° line, which means that model is sufficient for interpretation.

### Discussion and policy implications

The empirical results of the Random Forest algorithm can be summarized; the most important determinant affecting TLREF is the amount of securities bought by CBRT. Additionally, the emission amount, gold prices, USD/TL FX, XU100 index, the volatility index, CDS spreads, and the MSCI emerging market index are other influential determinants.

The analysis results reveal that global and country-level macroeconomic determinants influence TLREF. However, the COVID-19 pandemic does not influence TLREF for the period examined, contrary to initial expectations.

By considering the analysis results, it is proposed that Turkey should focus on eliminating the negative effects of country-level factors because it cannot lower the negative effects of global factors. In this context, the amount of securities bought by CBRT, country CDS spreads, USD/TL FX, amount of money issued by CBRT, and XU100 index can be better managed in Turkey’s different policies. For example, by decreasing the political risks of the country, CDS spreads can be decreased. Hence, foreign investment inflows to Turkey can be increased, and USD/TL FX can also be decreased. The USD/TL FX is quite significant for Turkey, as many FX-denominated debts affect the country’s credit rating notes, CDS spreads, and level of interest rates. Turkey can prevent the negative effects of FX on all economic indicators by keeping the USD/TL FX under control and at reasonable and fair levels. Additionally, similar effects can be provided with other policies such as comprehensive free trade agreements, swap agreements between Turkish Lira and convertible foreign exchange rates like USD, Euro, etc., decreasing the current account and foreign trade deficits.

After achieving success in managing country-level macroeconomic factors, Turkey should deal with global factors. In this context, Turkey can decrease the negative effects of global factors by eliminating the contagion effect. Specifically, stable FX rates should be provided for this aim.

Additionally, the critical thresholds in the effective variables should be considered. For example, TL 20 billion is a critical barrier for the number of securities bought by CBRT. In contrast, the barriers are 110,000 and 120,000 for the XU100 index, TL 150 billion for the amount of money issued by CBRT, and 350 for CDS spreads.

Moreover, the interactions between determinants should be considered in developing and applying policies. The analysis shows that there are interactions between gold prices and USD/TL FX; gold prices and amount of securities bought by CBRT; gold prices and amount of money issued by CBRT; USD/TL FX and amount of securities bought by CBRT; USD/TL FX and amount of money issued by CBRT; the number of securities bought by CBRT and amount of money issued by CBRT. These interactions reveal that a single variable should not be applied to any policy without considering the interaction with other determinants.

Besides the policies recommended above, other policies can be developed and applied by Turkish regulatory authorities. Possible outcomes of new policy practices on TLREF should be foreseen before implementation by considering each determinant’s threshold and interaction between determinants. These are several important issues that should be considered in policy development and application. Arguably, economic growth can be stimulated by developing the borrowing and securities issue capacity of Turkish financial institutions based on TLREF via increasing economic activities.

## Conclusion

LIBOR is retiring at the end of 2021, and countries should be ready to replace LIBOR with benchmark rates. In this context, the development of a national benchmark rate is required. As a new and financial innovation product, Turkey developed and announced TLREF as a benchmark rate. Financial institutions have used it to provide credits and issue bonds and bills based on TLREF. Hence, Turkey has been preparing for the replacement of LIBOR with TLREF. The study examines the development of TLREF and its main determinants since the beginning of the announcement.

The results and expected effects of determinants on TLREF are consistent except for that of oil prices. The results imply that Turkey should prioritize country-level macroeconomic determinants to have and sustain low-level TLREF because they are fully or mostly under the country’s control. In contrast, global factors cannot be controlled by Turkey. However, Turkey can decrease the negative effects of global factors on TLREF by eliminating the contagion effect of FX rates by making them stable. Additionally, some policy proposals are recommended based on the findings. The negative effects of the influential determinants on TLREF can be decreased, and economic activities and growth can be stimulated by increasing the borrowing and security issue capacity of Turkish banks on TLREF.

The main limitation of the study is that it focuses solely on Turkey. Examining multiple countries that announced the national benchmark rate, like the USA, UK, Euro Area, Switzerland, and Japan, could help expand the current literature. Additionally, other determinants can be included in a novel analysis; some determinants like economic growth, inflation, and unemployment cannot be considered due to the restriction on data available in the study. These determinants are announced monthly or quarterly, while TLREF is announced daily. When more data is accumulated, monthly or quarterly announced determinants could also be used. Moreover, other statistical and econometric methods like copula analysis, multivariate adaptive regression splines, neural networks, wavelet coherence approach, etc., can be applied for examining benchmark rates in the forthcoming studies.

## Data Availability

The data that support the findings of this study are available in Borsa İstanbul at https://www.borsaistanbul.com/veriler, Bloomberg Terminal, the Central Bank of the Republic of Turkey at https://evds2.tcmb.gov.tr/index.php?/evds/serieMarket, Turkish Ministry of Health at https://covid19.saglik.gov.tr.

## Appendix

See Table 3.

**Table 3:** BDS test results

Dimension	TLREF	VIX	GOLD	MSCI_EM	CDS	XU100	USDTL	CBRT_SEC	EMISSION
2	0.2008 [0.000]	0.1785 [0.000]	0.1956 [0.000]	0.1880 [0.000]	0.1823 [0.000]	0.1845 [0.000]	0.2000 [0.000]	0.2053 [0.000]	0.2031 [0.000]
3	0.3415 [0.000]	0.3036 [0.000]	0.3330 [0.000]	0.3180 [0.000]	0.3079 [0.000]	0.3120 [0.000]	0.33991 [0.000]	0.3471 [0.000]	0.3451 [0.000]
4	0.4397 [0.000]	0.3878 [0.000]	0.4292 [0.000]	0.4072 [0.000]	0.3928 [0.000]	0.3993 [0.000]	0.4375 [0.000]	0.4452 [0.000]	0.4439 [0.000]
5	0.5081 [0.000]	0.4431 [0.000]	0.4958 [0.000]	0.4681 [0.000]	0.4493 [0.000]	0.4584 [0.000]	0.5054 [0.000]	0.5136 [0.000]	0.5124 [0.000]
6	0.5550 [0.000]	0.4787 [0.000]	0.5416 [0.000]	0.5093 [0.000]	0.4854 [0.000]	0.4981 [0.000]	0.5524 [0.000]	0.5612 [0.000]	0.55593 [0.000]
Observations	945	945	945	945	945	945	945	945	945

The BDS test’s null hypothesis indicates that data in a time series is independently and identically distributed. The brackets show *p* values

**Authors’ information**

### **Dr. Özer Depren**

has been working as an Analysis and Decision Support Manager at Yapı Kredi Bank in İstanbul/Turkey for 10 years. He received the Ph.D. degree in Statistics from Marmara University, İstanbul/Turkey, 2014. His areas of interest are the Entropy, Performance Measuring Systems, Customer Experience Analytics, Advance Statistical Models, and Machine Learning Algorithms. Dr. Depren has more than 20 national/international publications in different journals indexed in SSCI, SCOPUS, and ESCI as well, and has authored 1 book chapter.

### **Dr. Mustafa Tevfik Kartal**

is a capital market professional. He received a Ph.D. degree in banking from Marmara University, İstanbul/Turkey in 2017, and received an Associate Professor degree in banking from Inter-Universities Board, Ankara/Turkey in 2020. His research interest focuses mainly on banking, central banking, economics, finance, capital markets, financial institutions, financial markets, and financial legislations. He has authored 1 book, 2 book reviews, 20 book chapters, 60 articles, and 12 proceedings in Turkish and English, some of which are indexed in SSCI, SCOPUS, and ESCI indices. Besides, his 4 articles have been accepted for publication and 11 articles have been under review. Also, he works on various articles related to CDS spreads, commodities, FX rates, interest rates, NPL, and stock market index currently. Moreover, he has a referee role in 53 national and international journals indexed in SSCI, SCOPUS, and ESCI as well.

### **Dr. Serpil Kılıç Depren**

works at Yildiz Technical University (YTU) Statistics Department, İstanbul/Turkey. She received the Ph.D. degree in Statistics from Marmara University in 2012. Her areas of interest include Hierarchical Linear Models, Generalized Estimation Equations, Mixed Models, Machine Learning Algorithms, Entropy, Data Analysis, and Multivariate Statistical Methods. Dr. Kılıç Depren has 36 international and 7 national publications in different journals indexed in SSCI, SCOPUS, and ESCI and has authored 1 book and 3 book chapter. Additionally, she has participated in 20 proceedings in international conferences and 4 proceedings in national conferences; as well, she won 7 academic awards from TUBITAK and YTU.

## Abbreviations

ARDL: Auto-Regressive Distributed Lag
BIST: Borsa İstanbul
BMR: Benchmark rates
CBRT: Central Bank of the Republic of Turkey
CDS: Credit default swap
CV: Coefficient of variation
ESTR: Euro Short Term Rate
FX: Foreign exchange rates
HIBOR: Hong Kong Interbank Offered Rate
LASSO: Least Absolute Shrinkage and Selection Operator
MAE: Mean Absolute Error
MSCI: Morgan Stanley Capital International
LIBOR: London Interbank Offered Rate
PDP: Public Disclosure Platform
OLS: Ordinary least squares
RMSE: Root Mean Square Error
SD: Standard Deviation
SIBOR: Singapore Interbank Offered Rate
SOFR: Secured Overnight Financing Rate
SONIA: Sterling Overnight Index Average
SARON: Swiss Average Rate Overnight
TLREF: Turkish Lira Overnight Reference Interest Rate
TONA: Tokyo Overnight Average Rate
USD: United States Dollar
VIX: Volatility index

## References

[CR1] Akkaya M (2018) Türk Lirası Referans Faiz Oranını (TRLIBOR) etkileyen makroekonomik faktörlerin analizi. Çankırı Karatekin Üniversitesi İİBF Dergisi 8(2):179–197

[CR2] Alexander C, Kaeck A (2008) Regime dependent determinants of credit default swap spreads. J Bank Finance 32(6):1008–1021

[CR4] BIS (2021) Beyond LIBOR: a primer on the new reference rates. https://www.bis.org/publ/qtrpdf/r_qt1903e.pdf. Accessed 16 Jan 2021

[CR5] BIST (2021a) TLREF. https://www.borsaistanbul.com/en/data/data/tlref-data. Accessed 16 Jan 2021

[CR6] BIST (2021b) Data. https://www.borsaistanbul.com/veriler. Accessed 16 Jan 2021

[CR7] Bloomberg (2021) Bloomberg terminal. Accessed 16 Jan 2021

[CR8] BoE (2021) SONIA key features and policies. https://www.bankofengland.co.uk/markets/sonia-benchmark/sonia-key-features-and-policies. Accessed 16 Jan 2021

[CR9] CBRT (2021) Electronic data distribution system. https://evds2.tcmb.gov.tr. Accessed 16 Jan 2021

[CR10] Chan FC (2011) An analysis of the relationship between choice of interest rate reference and interest rate risks of corporate borrowers. Doctoral dissertation, City University of Hong Kong

[CR11] Dinçer H, Yüksel S, Kartal MT (2019) The role of bank interest rate in the competitive emerging markets to provide financial and economic stability. J Econ Bus Finance Res 1(2):103–120

[CR12] Egert B, Crespo-Cuaresma J, Reininger T (2007) Interest rate pass-through in Central and Eastern Europe: reborn from ashes merely to pass away? J Policy Model 29:209–225

[CR13] EU (2016) Regulation 2016/1011 of the European parliament and of the council. https://eur-lex.europa.eu/legal-content/EN/TXT/PDF/?uri=CELEX:32016R1011&from=EN. Accessed 16 Jan 2021

[CR14] EU (2021a) Financial stability: commission addresses risks of Libor cessation. https://ec.europa.eu/commission/presscorner/detail/en/IP_20_1376. Accessed 16 Jan 2021

[CR15] EU (2021b) Review of the benchmark regulation. https://www.europarl.europa.eu/RegData/etudes/BRIE/2020/654187/EPRS_BRI(2020)654187_EN.pdf. Accessed 16 Jan 2021

[CR16] Galil K, Soffer G (2011) Good news, bad news and rating announcements: an empirical investigation. J Bank Finance 35(11):3101–3119

[CR17] Galil K, Shapir OM, Amiram D, Ben-Zion U (2014) The determinants of CDS spreads. J Bank Finance 41:271–282

[CR18] Goodell JW (2020) COVID-19 and finance: agendas for future research. Finance Res Lett 35:101512 10.1016/j.frl.2020.101512 PMC7152896 32562472

[CR19] Gopinathan R, Durai SRS (2019) Stock market and macroeconomic variables: new evidence from India. Financial Innov 5(1):1–17

[CR20] Gözgör G, Demir E, Belás J, Yeşilyurt S (2019) Does economic uncertainty affect domestic credits? An empirical investigation. J Int Financial Markets Inst Money 63:101147

[CR21] Gupta P, Goyal A (2015) Impact of oil price fluctuations on Indian economy. Energy Rev 39(2):141–162

[CR22] Hasan I, Liu L, Zhang G (2016) The determinants of global bank credit-default-swap spreads. J Financ Serv Res 50(3):275–309

[CR23] Hibbert AM, Pavlova I (2017) The drivers of sovereign cds spread changes: local versus global factors. Financ Rev 52(3):435–457

[CR24] Hol S (2006) Determinants of long-term interest rates in the Scandinavian countries. Norway Statistics Research Department Discussion Papers No. 469

[CR25] Holston K, Laubach T, Williams JC (2017) Measuring the natural rate of interest: international trends and determinants. J Int Econ 108:59–75

[CR26] ISDA (2021a) Adoption of risk-free rates: major developments in 2020. https://www.isda.org/a/WhXTE/Adoption-of-Risk-Free-Rates-Major-Developments-in-2020.pdf. Accessed 16 Jan 2021

[CR27] ISDA (2021b) Benchmark reform and transition from LIBOR. https://www.isda.org/2020/05/11/benchmark-reform-and-transition-from-libor. Accessed 16 Jan 2021

[CR28] Jammazi R, Ferrer R, Jareno F, Hammoudeh SM (2017) Main driving factors of the interest rate-stock market granger causality. Int Rev Financ Anal 52:260–280

[CR29] Johannes M, Sundaresan SM (2007) The impact of collateralization on swap rates. J Finance 62:383–410

[CR30] Kalgreen A (2019) Transitioning from LIBOR to a replacement rate index: what steps should lenders take now? J Equip Lease Financ 37(2):1–5

[CR31] Kanlı IB (2012) Which money market instrument is better at representing market expectations on short-term rates? CBRT Res Notes Econ 32:1–11

[CR32] Kartal MT (2019) Türkiye’de referans (gösterge) faiz oluşturulması: Türk Lirası gecelik referans faiz oranı (TLREF) üzerine bir inceleme. Bankacılar Dergisi 111:14–27

[CR33] Kartal MT (2019) Türkiye’de kredi faizlerini etkileyen faktörlerin belirlenmesi: MARS yöntemiyle bir analiz. Bankacılar Dergisi 108:24–41

[CR34] Kartal MT (2020) Determining affecting macroeconomic indicators on interest rates in emerging countries: a comparative examination upon China, Brazil, and Turkey with multivariate adaptive regression splines (MARS). J Empir Econ Soc Sci 2(1):23–41

[CR35] Kartal MT (2020) The behavior of sovereign credit default swaps (CDS) spread: evidence from Turkey with the effect of Covid-19 pandemic. Quant Finance Econ 4(3):489–502

[CR36] Kartal MT, Kılıç Depren S, Depren Ö (2018) Türkiye’de döviz kurlarını etkileyen makroekonomik göstergelerin belirlenmesi: MARS yöntemi ile bir inceleme. MANAS Sosyal Araştırmalar Dergisi 7(1):209–229

[CR37] Khun M, Johnson K (2013) Applied predictive modeling. Springer, New York

[CR38] Kim H, Shi W (2018) The determinants of the benchmark interest rate in China. J Policy Model 40(2):395–417

[CR39] Kou G, Peng Y, Wang G (2014) Evaluation of clustering algorithms for financial risk analysis using MCDM methods. Inf Sci 275:1–12

[CR40] Kou G, Chao X, Peng Y, Alsaadi FE, Herrera-Viedma E (2019) Machine learning methods for systemic risk analysis in financial sectors. Technol Econ Dev Econ 25(5):716–742

[CR41] Kou G, Xu Y, Peng Y, Shen F, Chen Y, Chang K, Kou S (2021) Bankruptcy prediction for SMEs using transactional data and two-stage multiobjective feature selection. Decis Support Syst 140:113429

[CR42] Kucheryavskiy S, Zhilin S, Rodionova O, Pomerantsev A (2020) Procrustes cross-validation—a bridge between cross-validation and independent validation sets. Am Chem Soc 92(17):11842–11850 10.1021/acs.analchem.0c02175 32786450

[CR43] Kumar M, Gupta DK, Singh S (2021) Extreme event forecasting using machine learning models. In: Hura G, Singh A, Siong Hoe L (eds) Advances in communication and computational technology. Lecture notes in electrical engineering 668. Springer, Singapore

[CR44] Law T, Shawe-Taylor J (2017) Practical bayesian support vector regression for financial time series prediction and market condition change detection. Quant Finance 17(9):1403–1416

[CR45] Lin X, Wang C, Wang N, Yang J (2018) Investment, Tobin’sq, and interest rates. J Financ Econ 130(3):620–640

[CR46] Lyashenko A, Mercurio F (2019) Looking forward to backward-looking rates: a modeling framework for term rates replacing LIBOR. Available at SSRN

[CR47] Maitra B (2017) Monetary and fiscal factors in nominal interest rate variations in Sri Lanka under a deregulated regime. Financ Innov 3(23):1–17

[CR48] MHT (2020) COVID-19 numbers. https://COVID19.saglik.gov.tr. Accessed 16 Jan 2021

[CR49] Mitchell K, Pearce DK (2017) Direct evidence on sticky information from the revision behavior of professional forecasters. South Econ J 84(2):637–653

[CR50] Muinhos MK, Nakane MI (2006) Comparing equilibrium real interest rates: different approaches to measure Brazilian rates. Banco Central do Brasil Discussion Paper No. 101

[CR51] Mukhtar T, Zakaria M (2008) Budget deficits and interest rates: an empirical analysis for Pakistan. J Econ Coop 29(2):1–14

[CR52] Obeng SK, Sakyi D (2017) Macroeconomic determinants of interest rate spreads in Ghana. Afr J Econ Manag Stud 8(1):76–88

[CR53] Onanuga AT, Shittu AM (2010) Determinants of interest rates in Nigeria: an error correction model. J Econ Int Finance 2(11):261–271

[CR54] Paramati SR, Gupta R (2013) An empirical relationship between exchange rates, interest rates and stock returns. Eur J Econ Finance Adm Sci 56:168–181

[CR55] Parmezan ARS, Souza VMA, Batista GAPA (2019) Evaluation of statistical and machine learning models for time series prediction: identifying the state-of-the-art and the best conditions for the use of each model. Inf Sci 484:302–337

[CR56] PDP (2021) https://www.kap.org.tr/en/bildirim-sorgu. Accessed 16 Jan 2021

[CR57] Ratti R, Vespignani JL (2016) Oil prices and global factor macroeconomic variables. Energy Econ 59:198–212

[CR58] Rizwan MS, Ahmad G, Ashraf D (2020) Systemic risk: the impact of COVID-19. Finance Res Lett 36:101682 10.1016/j.frl.2020.101682 PMC7334938 32837376

[CR59] Salim A (2019) Macroeconomic determinants of interest rate volatility in Indonesia: a structural var analysis. Int J Appl Econ Finance Account 5(2):101–108

[CR60] Schonlau M, Zou RY (2020) The random forest algorithm for statistical learning. Stata J Promot Commun Stat Stata 20(1):3–29

[CR61] Shapiro AF (2018) Labor force participation, interest rate shocks, and unemployment dynamics in emerging economies. J Dev Econ 133:346–374

[CR62] Shaukat B, Zhu Q, Khan MI (2019) Real interest rate and economic growth: a statistical exploration for transitory economies. Phys A 534:1–22

[CR63] Shen F, Zhao X, Kou G (2020) Three-stage reject inference learning framework for credit scoring using unsupervised transfer learning and three-way decision theory. Decis Support Syst 137:113366

[CR64] Taylor JB, Wieland V (2016) Finding the equilibrium real interest rate in a fog of policy deviations. Bus Econ 51(3):147–154

[CR65] Terzioğlu MK (2013) Gösterge faiz orani, diş ticaret hacmi ve iç borç stok ilişkisi. Akdeniz Üniversitesi İİBF Dergisi 26:55–76

[CR66] Tokic D (2018) Replacing LIBOR: Is BTFR the right choice? J Corp Account Finance 29(1):145–150

[CR67] Tumwine S, Sejjaaka S, Bbaale E, Kamukama N (2018) Determinants of interest rate in emerging markets: a study of banking financial institutions in Uganda. World J Entrep Manag Sustain Dev 14(3):267–290

[CR68] Tursoy T (2019) The interaction between stock prices and interest rates in Turkey: empirical evidence from ARDL bounds test cointegration. Financ Innov 5(7):1–12

[CR69] WHO (2021) Coronavirus disease (COVID-19) outbreak situation. https://www.who.int/emergencies/diseases/novel-coronavirus-2019. Accessed 16 Jan 2021

[CR70] Wong WK, Khan H, Du J (2006) Do money and interest rates matter for stock prices? An econometric study of Singapore and USA. Singap Econ Rev 51(1):31–51

[CR71] Yang L, Yang L, Hamori S (2018) Determinants of dependence structures of sovereign credit default swap spreads between G7 and BRICS countries. Int Rev Financ Anal 59:19–34

[CR72] Zhang S, Chen Y, Zhang W, Feng R (2021) A novel ensemble deep learning model with dynamic error correction and multi-objective ensemble pruning for time series forecasting. Inf Sci 544:427–445

